# Patients’ perceptions of their “most” and “least” important medications: a retrospective cohort study

**DOI:** 10.1186/1756-0500-5-619

**Published:** 2012-11-02

**Authors:** Amy Linsky, Steven R Simon

**Affiliations:** 1 Section of General Internal Medicine, VA Boston Healthcare System, 150 S. Huntington Ave, Boston, MA, 02130, USA; 2 Section of General Internal Medicine, Boston Medical Center, 801 Massachusetts Ave, Boston, MA, 02118, USA; 3 Boston University School of Medicine, Boston, USA; 4 Division of General Internal Medicine, Brigham and Women’s Hospital, Boston, USA; 5 Harvard Medical School, Boston, MA, USA

**Keywords:** Communication, Adherence, Veterans, Quality of care, Patient safety

## Abstract

**Background:**

Despite benefits of adherence, little is known about the degree to which patients will express their perceptions of medications as more or less important to take as prescribed. We determined the frequency with which Veteran patients would explicitly identify one of their medications as “most important” or “least important.”

**Findings:**

We conducted a retrospective cohort study of patients from ambulatory clinics at VA Boston from April 2010-July 2011. Patients answered two questions: “Which one of your medicines, if any, do you think is the most important? (if none, please write ‘none’)” and “Which one of your medicines, if any, do you think is the least important? (if none, please write ‘none’).” We determined the prevalence of response categories for each question. Our cohort of 104 patients was predominantly male (95%), with a mean of 9 medications (SD 5.7). Regarding their most important medication, 41 patients (39%) identified one specific medication; 26 (25%) selected more than one; 21 (20%) wrote “none”; and 16 (15%) did not answer the question. For their least important medication, 31 Veterans (30%) chose one specific medication; two (2%) chose more than one; 51 (49%) wrote “none”; and 20 (19%) did not directly answer the question.

**Conclusions:**

Thirty-five percent of patients did not identify a most important medication, and 68% did not identify a least important medication. Better understanding of how patients prioritize medications and how best to elicit this information will improve patient-provider communication, which may in turn lead to better adherence.

## Findings

### Background and objectives

Medication adherence may improve clinical outcomes, but approximately half of all prescriptions are not taken as prescribed [1-3]. Patient medication-taking behavior is influenced by many factors, including health literacy, socioeconomic status, perceived medication necessity, future health concerns and whether the drug provides symptom relief [4-6]. Further, patients’ beliefs about their medications are dynamic and can fluctuate with changes in symptoms, competing health- and non-health-related demands and trust in the health care provider [5,7].

Patient non-adherence appears to imply that some degree of prioritization of medications is occurring, although not necessarily in an explicit manner. Moreover, it is unclear to what degree patients will express their perceptions of medications as more or less important to their treating health care provider and whether clinicians concur with patients’ prioritization schemas. To begin to address these questions, we sought to determine the frequency with which Veteran patients would explicitly identify one of their medications as “most important” or “least important,” as well as to characterize the medications selected.

## Methods

We conducted a retrospective cohort study of a convenience sample of patients from ambulatory care clinics at Veterans Affairs (VA) Boston Healthcare System from April 2010 until July 2011. Patients were former members of the United States military who sought and were eligible to receive care at the VA. Data were collected by fourth-year medical students, who were individually instructed on project processes as part of their Ambulatory Medicine Quality Improvement rotation. One student per month was assigned to the rotation. Immediately prior to a student-led clinical encounter, patients were given a printout of the electronic health record (EHR) listing of their medications and asked to answer two questions: “Which one of your medicines, if any, do you think is the most important? (if none, please write ‘none’)” and “Which one of your medicines, if any, do you think is the least important? (if none, please write ’none’).” If needed, the student assisted the Veterans by reading the questions or writing their responses.

Because the data collection was intended as a quality improvement educational project, informed consent was not obtained. One of us (AL) entered all data into an Excel database, and we analyzed only the first chronologic encounter for each Veteran. Patient responses were entered exactly as written. Specific medications identified by patients were classified into medication classes using VA Drug Class Codes. Other data extracted from the EHR included patient sex, age at visit and number of actively prescribed medications.

Our two primary outcomes were patients’ responses to the “most important” and “least important” questions. Responses to each question were categorized as one of four types: 1) One specific medication – the patient identified one medication only; 2) More than one medication – the patient reported more than one medication, chose medications to treat a diagnosis (e.g. “heart meds”) or wrote “all”; 3) None – the patient wrote “none”; and 4) Did not answer the question – the patient left the response blank or wrote “n/a,” “don’t know,” “uncertain” or “not sure.”

We determined the prevalence of each primary outcome. Frequency counts identified the medication classes involved for responses where specific medications were chosen. Finally, we used chi-square tests to assess for associations between patient factors and choosing one specific medication as most or least important. All analyses were performed with SAS version 9.2 (SAS Institute, Inc) or Excel (Microsoft). Statistical significance was set at alpha < 0.05. This study protocol was approved by the Institutional Review Board of the VA Boston Healthcare System.

## Results

Our study cohort of 104 Veterans was predominantly male (95%), and 59 (57%) were age 65 years or older (Table 1). Lists contained a mean of 9 medications (SD 5.7). Responding to the question about their most important medication, 41 patients (39%) identified one specific medication; 26 (25%) selected more than one; 21 (20%) wrote “none”; and 16 (15%) did not answer the question (Table 2). Answering the question about their least important medication, 31 Veterans (30%) chose one specific medication; two (2%) chose more than one; 51 (49%) wrote “none”; and 20 (19%) did not directly answer the question (Table 2).

**Table 1:** Baseline characteristics (n=104)

**Patient characteristics**	**n (%)**
Age 65 or older	59 (57)
Male	99 (95)
Total number of medications [mean (SD)]	9 (5.7)

^*^Care may have occurred previously or concurrently with present care at VA Boston.

^†^Medications dispensed from other VA facilities (i.e., remotely-dispensed), non-VA medications or documented as inpatient medications.

**Table 2:** Categories of responses to identification of most and least important medication

**Response category**	**Most important n (%)**	**Least important n (%)**
One specific medication	41 (39)	31 (30)
More than one medication*	26 (25)	2 (2)
More than one medication	5 (5)	0 (0)
Chose medications for a condition (did not name a specific medication)	11 (11)	1 (1)
More than one medication and chose it by condition	3 (3)	0 (0)
Wrote “all”	7 (7)	1 (1)
Wrote “None”	21 (20)	51 (49)
Did not answer the question*	16 (15)	20 (19)
Wrote “n/a”	4 (4)	5 (5)
Left it blank	10 (10)	11(11)
Wrote “don’t know,” “uncertain,” or “not sure”	1 (1)	4 (4)
Wrote something undecipherable	1 (1)	0 (0)

*Subcategories sum to the total for their respective categories.

There was no association between selecting one medication as most or least important and any of the available patient factors (data not shown). The most commonly cited most important medication classes were beta blockers (n = 8), angiotensin converting enzyme inhibitors (n = 7) and anticoagulants (n = 5). The most commonly cited least important medication classes were vitamins (n = 12), non-opioid analgesics (n = 4), antilipemic agents (n = 2) and antigout agents (n = 2). Full results of medications classes identified by patients as most important and least important are shown in Table 3.

**Table 3:** Medication classes chosen as most or least important

**Most important medication**	n^*^
Beta blockers	8
Angiotensin converting enzyme inhibitors	7
Anticoagulants	5
Opioid analgesics	4
Calcium channel blockers	3
Non-opioid analgesics	2
Gastric medications	2
Digitalis glycosides	2
Antilipemic agents	2
Glucocorticoids	2
Insulin	2
Loop diuretics	2
Platelet aggregation inhibitors	1
Sedatives/hypnotics	1
Anticonvulsants	1
Antiparkinson agents	1
Antianginals	1
Thiazides/related diuretics	1
Local anesthetics, topical	1
Digestants	1
Genitourinary agents	1
Oral hypoglycemic agents	1
Antirheumatic agents	1
Skeletal muscle relaxants	1
Bronchodilators	1
**Least important medication**	**n**
Vitamins	12
Non-opioid analgesics	4
Antilipemic agents	2
Antigout agents	2
Sedatives/Hypnotics	1
Anticonvulsants	1
Loop diuretics	1
Angiotensin converting enzyme inhibitors	1
Local anesthetics, topical	1
Laxatives	1
Histamine antagonists	1
Antispasmodics, urological	1
Contraceptives, systemic	1
Skeletal muscle relaxants	1
Iron	1

^*^Unit of analysis is medication. Results do not include responses for condition-related medications (i.e., “heart meds”).

## Discussion

Approximately one in three Veteran patients did not identify a most important medication, and more than two in three patients did not identify a least important medication. There are several possible reasons to explain this finding. Patients may not have understood the questions, leading them to choose medications to treat a particular condition – therefore not selecting a single medication – or simply to leave the response blank. Limited health literacy, reflected as poor understanding of their health conditions or of their medications and associated indications, may have contributed to low response rates [8-10]. Without full understanding of their medical problems, the potential consequences of untreated health conditions and the possible benefits and risks of medication for those conditions, some patients may have been unable to make an informed selection from their medication list. This notion highlights the importance of education and knowledge in enabling patients to maintain an active role in their health care decisions.

Another hypothesis for the observed pattern of responses is that the use of the word “important” may not accurately reflect the construct that we were attempting to measure. That is, patients may have perceived medications as important but may not have believed that they are of a necessity or benefit to their future health to warrant full adherence. Others have demonstrated that perceived adverse effects of medications often outweigh preventive benefits of medications [11]. Competing demands, such as financial or social obligations, may further outweigh patient perceptions of importance. Psychometric testing of the best way to assess patient prioritization of medications with reliability and validity will improve measurement in future studies.

Another possible explanation for our findings is that Veteran patients were in fact able to understand and identify a medication yet they were unwilling to share these beliefs with their providers. Patients discontinue prescriptions for a variety of reasons without informing their healthcare provider of this decision [12]. Improving communication between patients and providers can lead to better shared decision making and better adherence [2].

Our study results need to be interpreted in the context of several limitations. We analyzed a small convenience sample of patients from one site within a larger health care system, and the study participants’ responses may not reflect the views of Veterans receiving care elsewhere or the perceptions of non-Veterans. Future research involving multiple settings and patient populations will enable better generalizability. We also had limited information on patients’ comorbidities and other medications, restricting us from appraising patient responses as concordant with clinician opinion. However, these answers still reflect what the patient perceived, and it is recognized that patient beliefs are associated with medication adherence [4]. Additionally, explicit discussions may enable providers to better reconcile known conflicts between what they believe is clinically best and what the patient perceives as important [13,14]. Finally, the training status of providers (i.e., medical students) theoretically could have influenced patients’ willingness to divulge their prioritization.

In this study population where the mean number of medications was nine, higher than most commonly accepted definitions of polypharmacy [15], a minority of patients were able to express a medication as least important. Better understanding of how patients prioritize their medications and how best to elicit this information will improve patient-provider communication and perhaps lead to discontinuation of medications that both the patient and the clinician feel have less importance.

## Abbreviations

VA: Veterans affairs; EHR: Electronic health record (EHR); SD: Standard deviation.

## Competing interests

The authors declare that they have no competing interests.

## Authors’ contributions

AL was responsible for study concept and design; acquisition, analysis and interpretation of data; and drafting and critical revision of the manuscript. SRS was involved in analysis and interpretation of data and critical revision of the manuscript. AL had full access to all of the data in the study and takes responsibility for the integrity and accuracy of the data analysis. All authors read and approved the final manuscript.
